# High temperature intensifies negative density dependence of fitness in red flour beetles

**DOI:** 10.1002/ece3.1402

**Published:** 2015-02-08

**Authors:** William D Halliday, Alison S Thomas, Gabriel Blouin-Demers

**Affiliations:** Department of Biology, University of OttawaOttawa, Ontario, Canada

**Keywords:** Oviposition, population growth, reproduction, thermal quality, *Tribolium castaneum*

## Abstract

Competition for food, space, or other depletable resources has strong impacts on the fitness of organisms and can lead to a pattern known as negative density dependence, where fitness decreases as population density increases. Yet, many resources that have strong impacts on fitness are nondepletable (e.g., moisture or temperature). How do these nondepletable resources interact with depletable resources to modify negative density dependence? We tested the hypothesis that negative density dependence is modulated by temperature in red flour beetles and tested the prediction that the strength of negative density dependence should decrease as temperature decreases. We measured the number of eggs laid, offspring development time, and the number of offspring that reached maturity at three temperatures and two food treatment combinations as we simultaneously manipulated adult population density. We demonstrated that low temperatures weaken negative density dependence in the number of eggs laid; this pattern was most evident when food was abundant. Density had no effect on development time, but low temperatures increased development time. The percent of eggs that emerged as adults decreased with both density and temperature and increased with food. Temperature, an abiotic driver, can thus modulate density-dependent processes in ectotherms. Therefore, models of population growth for ectotherms should incorporate the effects of temperature.

## Introduction

Competition is an important mechanism of ecological and evolutionary processes (e.g., Holt 1996; Travis et al. 1999; Brook and Bradshaw 2006; Morris 2011). Competition for food, space, or other depletable resources leads to negative density dependence, where fitness decreases with increasing density because as population density increases, competition increases, resource availability decreases, and, consequently, per capita fitness decreases. The strength of negative density dependence is therefore a function of the strength of competition (Sillett and Holmes 2005). When individuals require small quantities of an abundant depletable resource, competition for this resource is weak and density dependence is weak. Conversely, when a depletable resource is a limiting factor for an organism and is in short supply, competition is strong and negative density dependence is strong.

The abundance of depletable resources is rarely the sole limiting factor for organisms (Birch 1957; Hairston et al. 1960). Abiotic factors, such as moisture and temperature, can also be limiting factors for many organisms (Huey 1991). For example, moisture content is the principal limiting factor for many species, including fleas (*Xenopsylla* spp.; Krasnov et al. 2001), dung beetles (*Onthophagus* spp.; Sowig 1995), and red flour beetles (*Tribolium castaneum*; Howe 1956, 1962). Temperature is also a limiting factor for all ectotherms, as they rely on environmental temperature to maintain internal body temperature. Ectotherms select body temperatures that maximize several processes (Huey 1991), including metabolic rate (e.g., Gillooly et al. 2001), growth rate (e.g., Angilletta et al. 2004), locomotion (e.g., Blouin-Demers and Weatherhead 2008), and reproduction (Berger et al. 2008). Ectotherms do not generally compete for temperature per se, but competition for space can interfere with thermoregulation under the rare circumstances where very few microhabitats are at the ideal temperature, and the organism is territorial (e.g., Calsbeek and Sinervo 2002). As all ectotherms require food to survive, food is a limiting factor, but clearly temperature and food do not limit ectotherm populations independently.

We hypothesize that competition should be strongest, and hence, density dependence should be strongest, at high temperatures in ectotherms. Competition should be higher at high temperatures for at least two reasons: because the ability to harvest and process food increases with temperature and because metabolic rate, and hence energy demand, also increases with temperature (Dubois et al. 2008). In this study, we test this hypothesis by examining the effects of temperature, food abundance, and population density on fitness in red flour beetles (*Tribolium castaneum*) in a fully factorial design. The individual effects of temperature, food, and density on the fitness of flour beetles (both *T*. *castaneum* and *T. confusum*) have been studied for 90 years (Chapman 1924; Park 1932, 1934; Park and Frank 1948; Howe 1956, 1962; Taylor 1965; McDonald 1968; King and Dawson 1973; Lamb and Loschiavo 1981; Campbell and Runnion 2003). Nevertheless, the interactive effects of temperature, food, and density on fitness of *T*. *castaneum* have only recently been examined (Halliday and Blouin-Demers 2014). This is an important addition because, as explained above, we expect interactive effects to be present based on first principles. This study is therefore novel in its examination of how temperature modulates negative density dependence of fitness.

**Figure 1:**
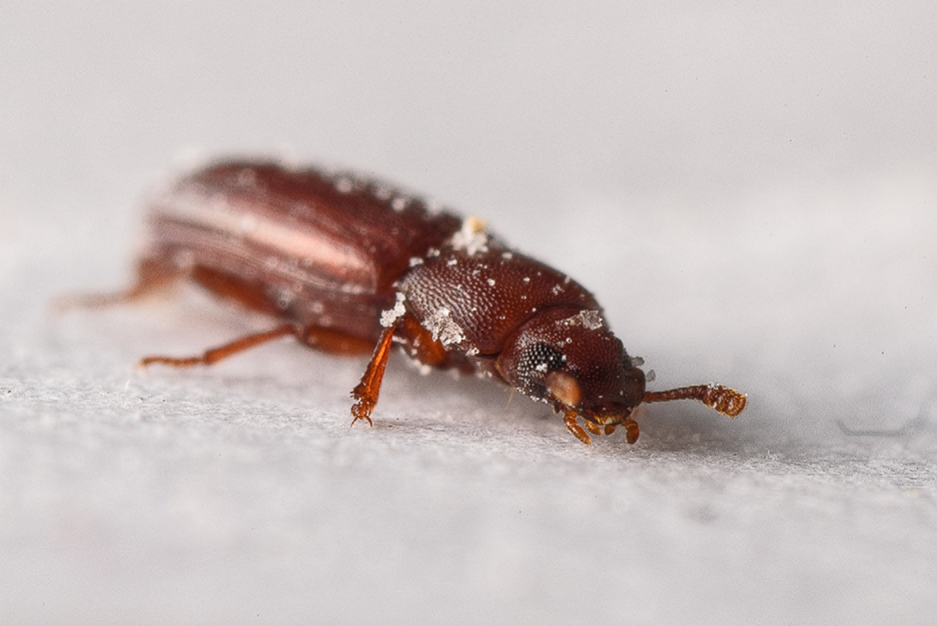
Photo of the red flour beetle (*Tribolium castaneum*). Photo credit: Antoine Morin.

## Materials and Methods

We conducted all experiments with a colony of red flour beetles (*Tribolium castaneum*) originally obtained from Carolina Biological Supply Company (Burlington, NC). The starting colony consisted of 200 individuals, and we let the colony grow to approximately 5000 individuals. We raised beetles in large cultures containing 95% all-purpose wheat flour and 5% brewer's yeast (all future mention of flour refers to this mixture). We maintained the cultures at 25°C and 70% humidity, with a 12 h light and 12 h dark photoperiod.

**Figure 2:**
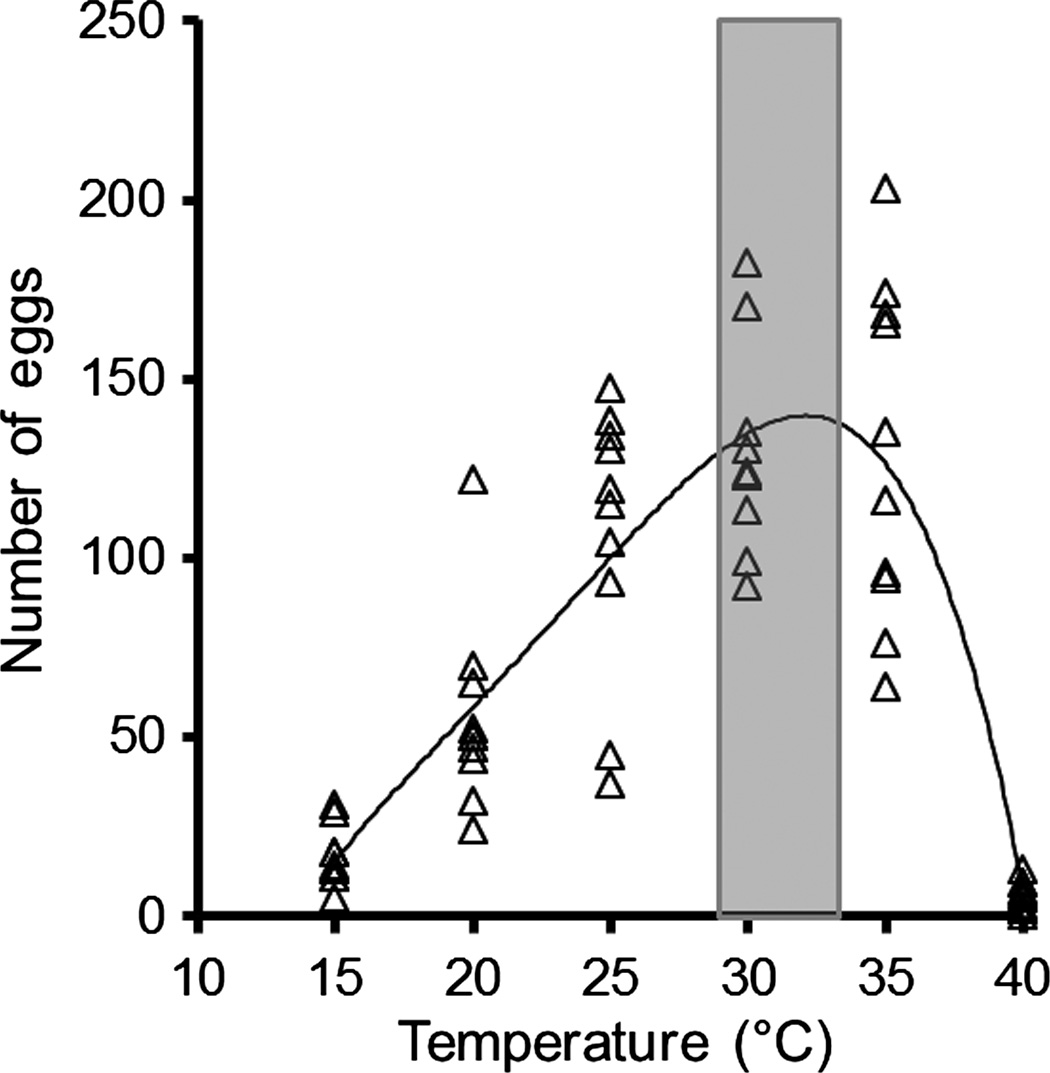
Number of eggs laid by 20 red flour beetles (*Tribolium castaneum*) over 4 days at different temperatures. *N *= 10 replicates for each temperature treatment. The line represents the quartic line of best fit. The gray box represents the interquartile range of selected temperatures by red flour beetles in a thermal gradient ranging from 20 to 40°C.

To ensure the ecological realism of our temperature treatments and to ensure there would be an effect of temperature, we determined the thermal reaction norm for oviposition by red flour beetles by measuring the number of eggs laid at 15, 20, 25, 30, 35, and 40°C (Fig.2). We placed 20 randomly selected adult beetles (assuming a 1:1 sex ratio; see below) into a petri dish (10 cm diameter) with 2.5 mL of flour and counted the number of eggs laid after 4 days. We replicated each temperature treatment 10 times. We used a quartic function to describe the relationship between temperature and the number of eggs laid and used the maximum of this function (29.6°C, rounded to 30°C) as the optimal temperature for egg laying (Fig.2).

We then tested the effects of temperature (20, 25, and 30°C), food abundance (0.625 and 2.5 mL of flour), and population density (10, 20, 30, 40, and 50 beetles) on fitness in a fully factorial design, with five replicates of each treatment combination. We used 20, 25, and 30°C as temperature treatments based on their effect on oviposition rate (Fig.2). In addition, 30°C is the preferred temperature of red flour beetles in a laboratory thermal gradient (Halliday and Blouin-Demers 2014). For each replicate, we placed adult beetles (0–3 months old), randomly selected from our large colony, in a petri dish (10 cm diameter) with flour that had been sieved through a 250 *μ*m sieve, placed the petri dish in an incubator, and allowed the adults to lay eggs for four days. We then removed the adults, sifted through the flour with a 250 *μ*m sieve, and counted the number of eggs. We replaced the used flour with fresh flour, placed the eggs back in the petri dish, and put the dish back in the incubator. We replaced the used flour with fresh flour weekly until all offspring were adults or all larvae had died. Eggs in the 20°C treatment did not develop; therefore, we only use data from this treatment for the comparison of oviposition rates.

We analyzed the per capita number of eggs (# eggs/density treatment, square-root transformed) and the mean time to the emergence of adult offspring using linear regression in R (package: stats; function: lm; R Core Team 2012) with temperature, quantity of food, density, and all two- and three-way interactions as independent variables. We used bias-corrected Akaike's information criteria (package: qpcR; function: AICc; Spiess 2012) to select the best model. Models with ΔAICc < 2 were considered competing models (Burnham and Anderson 2002), and we selected the competing model with the fewest parameters as the final model if all the terms in the other competing models were nonsignificant (Bozdogan 1987).

We also analyzed the per capita number of offspring reaching maturity (# adult offspring/density treatment) and the proportion of eggs that developed into adults (# adult offspring/# eggs) using generalized linear models with a quasi-Poisson distribution in R (package: stats; function: glm; family: quasi-Poisson), with the same independent variables as the previous analyses. We used a quasi-Poisson distribution because analyses with a Poisson distribution were overdispersed, and the data could not be transformed to fit a normal distribution. We analyzed the proportion of eggs that developed into adults because the number of adult offspring was correlated with the number of eggs laid at lower densities (see below); therefore, this analysis allowed us to distinguish between more eggs laid leading to a higher number of adult offspring and the ability of eggs to develop into adults. We used deviance analysis with a chi-squared test (package: stats; function: anova; test: chi-squared) to select the final terms in each generalized linear model.

We examined the relationship between the number of eggs laid and the number of eggs that survived to the adult stage, while controlling for density, temperature, and quantity of food using a generalized linear model with a quasi-Poisson distribution. We used the number of adult offspring as the dependent variable, with the number of eggs laid, density, temperature, quantity of food, and all two-way interactions between the number of eggs laid and the other variables as independent variables. We used deviance analysis with a chi-squared test to select the final terms of the model.

## Results

**Figure 3:**
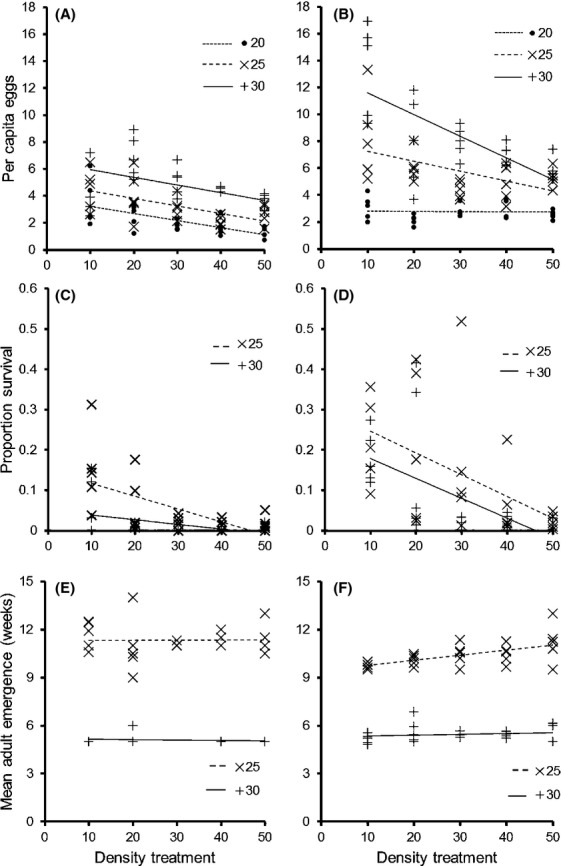
Per capita number of eggs (number of eggs/number of adults) laid over 4 days (A, B), the proportion of eggs that emerged as adults (Proportion Survival; C, D), and the mean time to emergence of adult offspring (Mean Adult Emergence; E, F) by red flour beetles (*Tribolium castaneum*) at different temperatures (20, 25, and 30°C) and population densities. Treatments either had low food (0.625 mL flour; A, C, E) or high food (2.5 mL flour; B, D, F). *N *= 5 replicates for each treatment combination. The lines represent the linear lines of best fit for each treatment. Treatments at 20°C did not develop past the egg stage.

For the per capita number of eggs laid, negative density dependence was strongly modulated by temperature in the high food treatment, but not in the low food treatment (full model *R*^2 ^= 0.73; Fig.3; Table S1). In the high food treatment, there was no density dependence at 20°C (slope ± SE = −0.0001 ± 0.002, *t*_1,23 _= 0.04, *P *=* *0.97), some negative density dependence at 25°C (slope ± SE = −0.01 ± 0.005, *t*_1,23 _= 2.81, *P *<* *0.01), and strong negative density dependence at 30°C (slope ± SE = −0.03 ± 0.006, *t*_1,23 _= 4.35, *P *<* *0.001). In the low food treatment, the per capita number of eggs increased with temperature (slope ± SE = 0.07 ± 0.009, *t*_1,72 _= 8.14, *p *<* *0.0001) and decreased with density (slope ± SE = −0.01 ± 0.002, *t*_1,72 _= 5.62, *p *<* *0.0001), but the slope of the relationship with density was unaffected by temperature (*t*_1,71 _= 0.68, *P *=* *0.50).

The mean time to emergence of adult offspring was unaffected by density (slope ± SE = 0.01 ± 0.006, *t*_1,72 _= 1.85, *P *=* *0.07; Fig.3, Table S2), but decreased markedly as temperature increased (mean time for 25°C ± SE = 10.82 ± 0.15 weeks; 30°C = 5.34 ± 0.08 weeks; *t*_1,73 _= 15.86, *P *<* *0.0001) and decreased more markedly as food increased at 25°C than at 30°C (temperature × food interaction: *t*_1,73 _= 3.29, *P *<* *0.01).

The per capita number of offspring produced that reached maturity was unaffected by temperature (slope ± SE = −0.03 ± 0.03, *t*_1,96 _= 0.93, *P *=* *0.35; Fig.3, Table S3), but increased with food (slope ± SE = 1.02 ± 0.13, *t*_1,97 _= 7.68, *p *<* *0.0001) and decreased with density (slope ± SE = −0.08 ± 0.008, *t*_1,97 _= 9.58, *P *<* *0.0001). The proportion of eggs that survived to the adult stage decreased with density (slope ± SE = −0.06 ± 0.009, *t*_1,96 _= 6.50, *P *<* *0.0001; Fig.3, Table S4), decreased with temperature (slope ± SE = −0.14 ± 0.04, *t*_1,96 _= 3.19, *P *<* *0.01), and increased with food (slope ± SE = 0.62 ± 0.13, *t*_1,96 _= 4.75, *p *<* *0.0001). The number of eggs that emerged as adults increased with the number of eggs laid in the lowest density treatment, but decreased with the number of eggs laid in all other density treatments (eggs: density interaction; *t *=* *2.36, *P *=* *0.02; Table S5). More eggs emerged as adults as food increased (*t *=* *2.09, *P *=* *0.04).

## Discussion

Fitness of red flour beetles, as measured by their oviposition rate and their number of offspring reaching maturity, was strongly modulated by temperature, indicating that temperature may affect competition for resources. While negative density dependence was strong at 30°C, it was reduced at 25°C and disappeared at 20°C, which suggests that competition for food decreases as temperature decreases. This supports our hypothesis that competition for food will be strongest at high temperature because, in ectotherms, the ability to process resources and their metabolic demands are a function of body temperature (Dubois et al. 2008). The absence of density dependence at 20°C indicates that there was no competition for food, probably because the ability to ingest and process food and the metabolic demands are much reduced at this low temperature; temperature was the rate limiting factor for fitness. This relationship between temperature and competition is likely caused by the effect of temperature on metabolism (e.g., Gillooly et al. 2001; Dubois et al. 2008), where low temperatures cause a reduction in metabolic rate, which then causes a reduction in the energy processing rate of individuals. Reduced rates of energy assimilation per individual then cause reduced demand for food, and therefore reduced competition. Reduced energetic requirements also make less energy available for growth and reproduction, therefore causing lower fecundity and longer development of larva.

We found no negative density dependence in development time, likely because the effect of density was masked by the effect of temperature: Temperature caused a difference of four weeks in development time while food only caused a difference of one week. This suggests that temperature could affect fitness in two ways: by modifying the number of offspring produced, but also by modifying how long it takes for these offspring to mature and reproduce. This could have repercussions at the population level. For example, ten adult beetles at 25°C with 2.5 mL of flour per week could lay 80 eggs in 4 days, which would then take nine weeks to develop into 18 new adult beetles, yielding an adult population of 28 beetles and a population growth rate of two adult beetles per week. Meanwhile at 30°C with 2.5 mL of flour per week, ten adult beetles could lay 140 eggs in 4 days, which would take five weeks to develop into 25 new adults, yielding an adult population of 35 beetles and a population growth rate of five adult beetles per week. In this example, based on our empirical results, population growth rate at 30°C is more than double that at 25°C.

Both the per capita number of eggs that emerged as adults and the proportion of eggs that emerged as adults increased with food abundance and decreased with density. This demonstrates that density has a negative impact on fitness at multiple life stages and confirms strong negative density dependence in this species. Temperature, on the other hand, had no effect on the per capita number of eggs that emerged as adults, but caused a decrease in the proportion of eggs that emerged as adults. The per capita number of eggs that emerged as adults did not control for the total number of eggs laid, whereas the proportion of eggs that emerged as adults is calculated based on the total number of eggs laid. Because the number of eggs laid increases with temperature, but the number of adult offspring is unaffected by temperature, the proportion of eggs that emerged as adults decreases with temperature.

The relationship between the number of eggs laid and the number of eggs that emerged as adults was only positive in the lowest density treatment. Overall, therefore, temperature had little influence on the number of resulting adult offspring. Beetles seem to maximize fitness differently as temperature varies: they maximize oviposition rate at high temperatures and maximize survival at low temperatures, with both strategies leading to a similar number of adult offspring.

We considered the effects of food abundance, density, and temperature on population growth of flour beetles. Other variables, such as egg cannibalism, also have large effects on population growth in flour beetles (Rich 1956; Sonleitner 1961; Taylor 1965). Future work could examine the effects of egg cannibalism on demography across multiple temperatures; egg cannibalism is likely also temperature and density dependent. Future work could also use a demographic modeling approach, along with more precise measures of larval competition and population growth rate, to model the effect of temperature on population dynamics in this species. This would help determine how temperature can affect density-dependent processes.

Negative density dependence is an important assumption in many ecological models, including demographic models (e.g., Lotka-Volterra equation [Volterra 1926], Ricker equation [Ricker 1954]), theories of habitat selection (e.g., Fretwell and Lucas 1969; Rosenzweig 1981), and in evolutionary models, including niche evolution (Holt 1996) and the evolution of dispersal (Travis et al. 1999). Abiotic factors, including temperature, can modulate negative density dependence in ectotherms (Birch 1957). Depending on the magnitude of their direct and interactive effects, abiotic factors may therefore also affect the general applicability of these ecological models to ectotherms.

## Supporting Information

**Appendix S1.** Model selection and final models for all analyses. **Table S1.** Model selection and final model for models examining the effect of temperature, density, and food on the per capita number of eggs laid by red flour beetles (*Tribolium castaneum*) over a four day period (sqrt-transformed). **Table S2.** Model selection and final model for models examining the effect of temperature, density, and food on the mean development time (time from egg to adult) of red flour beetles (*Tribolium castaneum*). **Table S3.** Analysis of deviance and final model for models examining the effect of temperature, density, and food on the per capita number of adult red flour beetle (*Tribolium castaneum*) offspring using generalized linear models with a quasi-Poisson distribution. **Table S4.** Analysis of deviance and final model for models examining the effect of temperature, density, and food on the proportion of red flour beetle (*Tribolium castaneum*) eggs that emerged as adults using generalized linear models with a quasi-Poisson distribution. **Table S5.** Model selection and final model for models examining the relationship between the number of eggs laid and the number of adult offspring red flour beetles (*Tribolium castaneum*), while controlling for the effects of temperature, density, and food.
