# Supplementary material for: High temperature intensifies negative density dependence of fitness in red flour beetles
Source: Ecol Evol. 2015 Feb 8;5(5):1061–7. doi: 10.1002/ece3.1402 (PMC4364820; doi:10.1002/ece3.1402)
Supplement: Supplementary file 1 [file ece30005-1061-sd1.docx]

Appendix – Model selection and final models for all analyses.

Table S1. Model selection and final model for models examining the effect of temperature, density, and food on the per capita number of eggs laid by red flour beetles (*Tribolium castaneum*) over a four day period (sqrt-transformed). The bolded model represents the best model according to AIC, while taking the number of parameters (*k*) into account.

| Model | | | *k* | AIC | ΔAIC |
| --- | --- | --- | --- | --- | --- |
| **Eggs = Temperature + Food + Density + Temperature : Food + Temperature : Density + Food : Density**  **+ Temperature : Food : Density** | | | **9** | **101.57** | **0.00** |
| Eggs = Temperature + Food + Density + Temperature : Food + Temperature : Density + Food : Density | | | 8 | 110.08 | 8.51 |
| Eggs = Temperature + Food + Density | | | 5 | 120.10 | 18.53 |
| Parameter | Estimate | S.E. | | *t* | *p* |
| Intercept | 1.70 | 0.76 | | 2.25 | 0.03 |
| Temperature | 0.01 | 0.03 | | 0.49 | 0.62 |
| Food | -1.57 | 0.42 | | 3.78 | < 0.0001 |
| Density | -0.05 | 0.02 | | 2.34 | 0.03 |
| Temperature : Food | 0.07 | 0.02 | | 4.43 | < 0.0001 |
| Temperature : Density | 0.001 | 0.0009 | | 1.60 | 0.11 |
| Food : Density | 0.04 | 0.01 | | 3.26 | 0.001 |
| Temperature : Food : Density | -0.001 | 0.0005 | | 3.25 | 0.001 |

Table S2. Model selection and final model for models examining the effect of temperature, density, and food on the mean development time (time from egg to adult) of red flour beetles (*Tribolium castaneum*). The bolded model represents the best model according to AIC, while taking the number of parameters (*k*) into account.

| Model | | | *k* | AICc | ΔAICc |
| --- | --- | --- | --- | --- | --- |
| Time = Temperature + Food + Density + Temperature : Food + Temperature : Density + Food : Density  + Temperature : Food : Density | | | 9 | 185.65 | 1.55 |
| Time = Temperature + Food + Density + Temperature : Food + Temperature : Density + Food : Density | | | 8 | 184.16 | 0.06 |
| Time = Temperature + Food + Density + Temperature : Food + Food : Density | | | 7 | 184.10 | 0.00 |
| Time = Temperature + Food + Density + Temperature : Food | | | 6 | 184.51 | 0.41 |
| **Time = Temperature + Food + Temperature : Food** | | | **5** | **185.81** | **1.71** |
| Parameter | Estimate | S.E. | | *t* | *p* |
| Intercept | 44.87 | 2.23 | | 20.12 | < 0.0001 |
| Temperature | -1.33 | 0.08 | | 15.86 | < 0.0001 |
| Food | -3.86 | 1.10 | | 3.51 | < 0.001 |
| Temperature : Food | 0.13 | 0.04 | | 3.29 | < 0.01 |

Table S3. Analysis of deviance and final model for models examining the effect of temperature, density, and food on the per capita number of adult red flour beetle (*Tribolium castaneum*) offspring using generalized linear models with a quasi-Poisson distribution. The bolded terms represent those selected for the final model according to analysis of deviance.

| Model | Residual *df* | Deviance | Residual Deviance | *p* | |
| --- | --- | --- | --- | --- | --- |
| Intercept | 99 | - | 95.71 | - | |
| Temperature | 98 | 0.270 | 95.44 | 0.60 | |
| **Food** | **97** | **27.70** | **67.75** | **< 0.0001** | |
| **Density** | **96** | **40.03** | **27.72** | **< 0.0001** | |
| Temperature : Food | 95 | 1.61 | 26.11 | 0.20 | |
| Temperature : Density | 94 | 1.65 | 24.46 | 0.20 | |
| Food : Density | 93 | 0.48 | 23.99 | 0.49 | |
| Temperature : Food : Density | 92 | 0.04 | 23.95 | 0.85 | |
| Parameter | Estimate | S.E. | *t* | | *p* |
| Intercept | -0.80 | 0.31 | 2.58 | | 0.01 |
| Food | 0.03 | 0.004 | 8.11 | | < 0.0001 |
| Density | -0.003 | 0.0003 | 9.88 | | < 0.0001 |

Table A4. Analysis of deviance and final model for models examining the effect of temperature, density, and food on the proportion of red flour beetle (*Tribolium castaneum*) eggs that emerged as adults using generalized linear models with a quasi-Poisson distribution. The bolded terms represent those selected for the final model according to analysis of deviance.

| Model | Residual *df* | Deviance | Residual Deviance | | *p* |
| --- | --- | --- | --- | --- | --- |
| Intercept | 99 | - | 13.12 | | - |
| **Temperature** | **98** | **0.84** | **12.28** | | **0.001** |
| **Food** | **97** | **2.06** | **10.22** | | **< 0.0001** |
| **Density** | **96** | **4.00** | **6.22** | | **< 0.0001** |
| Temperature : Food | 95 | 0.14 | 6.09 | | 0.20 |
| Temperature : Density | 94 | 0.19 | 5.90 | | 0.13 |
| Food : Density | 93 | 0.15 | 5.75 | | 0.18 |
| Temperature : Food : Density | 92 | 0.002 | 5.75 | | 0.87 |
| Parameter | Estimate | S.E. | | *t* | *p* |
| Intercept | 1.49 | 1.23 | | 1.22 | 0.23 |
| Temperature | -0.14 | 0.04 | | 3.19 | < 0.01 |
| Food | 0.62 | 0.13 | | 4.75 | < 0.0001 |
| Density | -0.06 | 0.009 | | 6.50 | < 0.0001 |

Table A5. Model selection and final model for models examining the relationship between the number of eggs laid and the number of adult offspring red flour beetles (*Tribolium castaneum*), while controlling for the effects of temperature, density, and food . The bolded model represents the best model according to AIC, while taking the number of parameters (*k*) into account.

| Model | Residual *df* | Deviance | Residual Deviance | *p* | |
| --- | --- | --- | --- | --- | --- |
| Intercept | 99 | - | 1376.20 | - | |
| **Eggs** | **98** | **20.02** | **1356.18** | **0.07** | |
| **Density** | **97** | **200.70** | **1155.48** | **< 0.0001** | |
| **Temperature** | **96** | **74.54** | **1080.95** | **< 0.001** | |
| **Food** | **95** | **490.18** | **590.77** | **< 0.0001** | |
| **Eggs : Density** | **94** | **24.03** | **566.74** | **0.048** | |
| Eggs : Temperature | 93 | 0.68 | 566.06 | 0.74 | |
| **Eggs : Food** | **92** | **23.96** | **542.11** | **0.048** | |
| Parameter | Estimate | S.E. | *t* | | *p* |
| Intercept | 1.40 | 1.43 | 0.98 | | 0.23 |
| Eggs | -0.02 | 0.01 | 1.68 | | 0.10 |
| Density | 0.03 | 0.02 | 1.48 | | 0.14 |
| Temperature | -0.009 | 0.04 | 0.21 | | 0.83 |
| Food | 0.71 | 0.34 | 2.09 | | 0.04 |
| Eggs : Density | -0.0003 | 0.0001 | 2.36 | | 0.02 |
| Eggs : Food | 0.007 | 0.004 | 1.81 | | 0.07 |
